# Transforming spinal surgery with innovations in biologics and additive manufacturing

**DOI:** 10.1016/j.mtbio.2025.101853

**Published:** 2025-05-13

**Authors:** Xinggui Tian, Yakui Liu, Suihong Liu, Qinyu Tian, Deepak Bushan Raina, Michael Gelinsky, Stefan Zwingenberger

**Affiliations:** a University Center of Orthopaedic, Trauma and Plastic Surgery, University Hospital Carl Gustav Carus at TUD Dresden University of Technology, Dresden, 01307, Germany; b Center for Translational Bone, Joint and Soft Tissue Research, University Hospital Carl Gustav Carus at TUD Dresden University of Technology, Dresden, 01307, Germany; c Engineering Science and Mechanics Department, Penn State University, University Park, 16802, PA, USA; d The Huck Institutes of the Life Sciences, Penn State University, University Park, 16802, PA, USA; e Department of Orthopaedics and Traumatology, Faculty of Medicine, The Chinese University of Hong Kong, Hong Kong Special Administrative Region of China; f Department of Clinical Sciences Lund, Orthopaedics, Faculty of Medicine, Lund University, Lund, 22185, Sweden

**Keywords:** Additive manufacturing, Biologics, Regenerative medicine, Spine surgery

## Abstract

The synergistic integration of biologics and additive manufacturing (AM) technologies has catalyzed groundbreaking advancements in spinal surgery. These innovations address persistent challenges, such as optimizing fusion outcomes, enhancing tissue regeneration, and achieving precise anatomical compatibility. Biologics have transformed spinal fusion by facilitating bone formation and promoting osteointegration through refinements in traditional materials and cutting-edge biologic developments. Concurrently, AM technologies, including 3D printing and biofabrication, enable the design and production of patient-specific implants and bioengineered scaffolds, significantly enhancing surgical precision and improving treatment outcomes. This review underscores the transformative synergy of biologics and AM, offering a comprehensive exploration of their applications in preclinical research and clinical practice. Together, these synergistic advancements are redefining the field of spinal surgery, driving the evolution of personalized and innovative treatment paradigms.

## Graphical abstract

**Figure:**
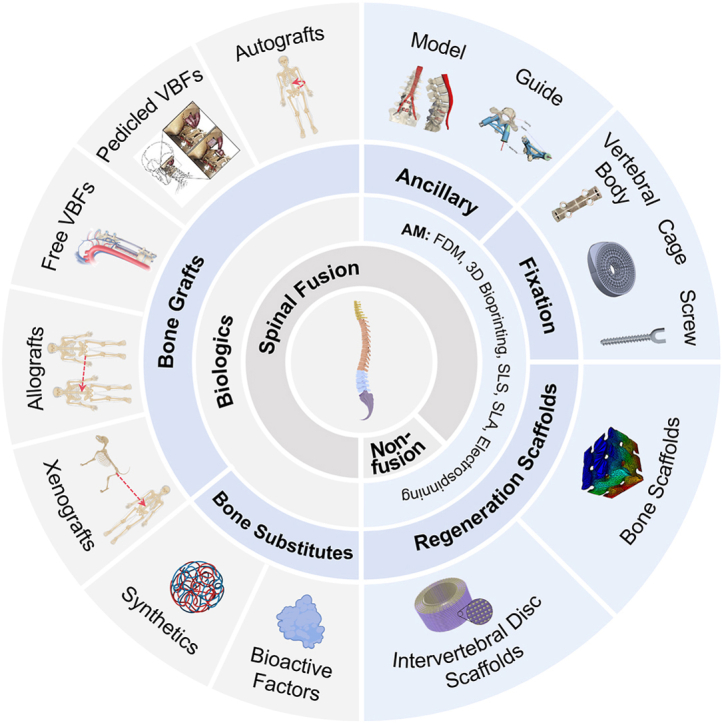

## 1 Introduction

The spine, also known as the vertebral column or backbone, is a fundamental component of the human axial skeleton, functioning as a dynamic, load-bearing structure that supports the trunk and limbs while providing vital protection for the spinal cord [1]. Spinal disorders, commonly manifesting as low back or neck pain, are among the leading causes of disability worldwide and pose a significant public health and economic burden. According to the latest Global Burden of Disease study, low back pain affected 619 million people worldwide in 2020, with projections indicating an increase to 843 million by 2050, remaining the leading cause of years lived with disability globally [2]. Similarly, 203 million people suffered from neck pain in 2020, with the number expected to reach 269 million by 2050, ranking 11th in the years lived with disability globally [3]. The rise in these cases is primarily due to population growth and aging [2,3]. Spinal disorders are grossly categorized into pain syndromes, deformities, degenerative diseases, infectious or inflammatory diseases, fractures, tumors, and osteoporosis [4], presenting significant economic challenges to the healthcare systems [5]. When conservative treatments fail to alleviate symptoms or restore function, surgical intervention becomes necessary to correct anatomical abnormalities, stabilize the spine, or decompress neural elements [5]. Advances in spinal surgery have been propelled by innovations in implant technology, biologic therapies, and emerging biomanufacturing techniques.

Spinal fusion remains a cornerstone of surgical management for many spinal pathologies, involving the permanent joining of two or more vertebrae to eliminate pathological motion [6,7]. However, fusion failure, referred to as pseudarthrosis, remains a major complication, often necessitating revision surgery [8]. The incidence of pseudarthrosis varies by spinal region: cervical fusion (2–30 %) [9], thoracic fusion (∼1.8 %) [10], and lumbar fusion (5–35 %) [11]. Pseudarthrosis can significantly compromise patient outcomes, leading to persistent pain, functional impairment, and psychological distress [8]. Therefore, enhancing fusion rates and reducing pseudarthrosis are critical imperatives in contemporary spinal surgery. To address these challenges, various bone grafting approaches have been employed, including autografts, allografts, xenografts, and synthetic bone substitutes.

In parallel, internal fixation devices such as pedicle screws, cages, rods, and artificial vertebral bodies (AVBs) are used to provide immediate structural stability. However, standard implants may not precisely match patient-specific anatomy, potentially limiting surgical efficacy [12]. Additive manufacturing (AM), commonly referred to as 3D printing, has emerged as a transformative tool in spinal surgery. AM enables the design and production of patient-specific implants and surgical guides tailored to complex anatomical structures, improving implant fit, surgical precision, and outcomes. Moreover, the integration of biomanufacturing, termed bio-additive manufacturing or biofabrication, has expanded the scope of AM to include bioprinting, advanced drug delivery systems, and tissue-engineered scaffolds [13,14]. These technologies hold promise in promoting spinal fusion, enabling functional tissue regeneration, and enhancing biological integration.

Although spinal fusion is effective in stabilizing the spine and alleviating pain, it inherently restricts motion at the fused segment. Moreover, fusion alters spinal biomechanics, increasing stress on adjacent segments, which can accelerate adjacent segment degeneration and disease, with reported incidence rates of 26.6 % and 8.5 % after lumbar fusion, and 32.8 % and 6.3 % after cervical fusion, respectively [15]. Consequently, the field has witnessed a shift toward non-fusion alternatives, aiming to preserve the mobility and function of the spine while alleviating the pain caused by intervertebral disc (IVD) damage, often employing artificial IVDs and nuclei replacements [5]. Ongoing advances in biomaterials science and regenerative medicine are driving the evolution of artificial IVDs from inert metal implants to bioengineered regenerative scaffolds, while biofabrication techniques are being used to better replicate the structure and function of the natural intervertebral disc.

This review provides a comprehensive examination of recent advancements in biologics and biofabrication technologies for spinal applications, synthesizing evidence from the past five years. It highlights current challenges, emerging opportunities, and potential pathways for innovation. By fostering interdisciplinary collaboration among spine surgeons, biomaterial scientists, and tissue engineers, this review aims to catalyze future breakthroughs in spinal repair and regeneration.

## 2 Spinal and intervertebral disc anatomy

**Fig. 1:**
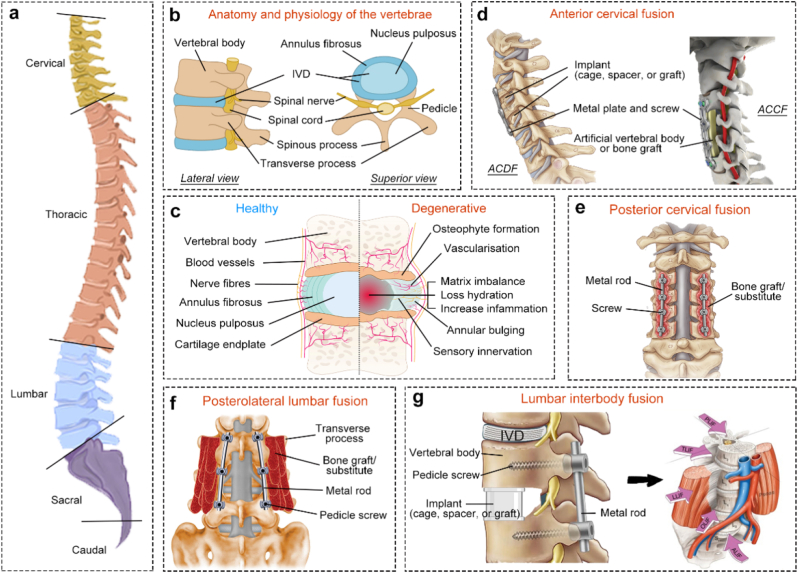
**(a**–**c)**. Anatomical view of the spine and **(d**–**g)** schematic of common cervical and lumbar fusion procedures. **(a)**. Lateral view of the whole spine and **(b)** anatomy and physiology of the vertebrae [22]. **(c)**. Graphical comparison of healthy and degenerated intervertebral disc (IVD) [18]. **(d**–**e).** Clinical application of common cervical fusion surgical approaches, **(d)** anterior cervical fusion [including anterior cervical discectomy and fusion (ACDF) [23] and anterior cervical corpectomy and fusion (ACCF) [24] and **(e)** posterior cervical fusion (PCF) [23]. **(d**–**e).** Clinical application of common lumbar fusion surgical approaches, **(f)** posterolateral lumbar fusion (PLF) [25] and **(g)** lumbar interbody fusion (LLF) [26] [including anterior (ALIF), lateral or extreme lateral (LLIF or XLIF), oblique (OLIF), transforaminal (TLIF), and posterior (PLIF) lumbar interbody fusion] [21].

The spine is composed of individual bones called vertebrae, which are segmented into five regions: cervical, thoracic, lumbar, sacral, and caudal. The human spine is composed of 32–33 vertebrae at birth, while in adulthood the spine usually consists of 26 vertebrae due to physiological fusion evolution, including 7 cervical, 12 thoracic, 5 lumbar, 1 sacral (formed by the fusion of 5 sacral vertebrae), and 1 caudal (formed by the fusion of 3–4 caudal vertebrae) [16,17] (Fig. 1a). These vertebrae are interconnected by IVDs, except for the first and second cervical vertebrae [16,17]. The IVD is a fibrocartilaginous tissue that consists of a central core, proteoglycan-rich gelatinous nucleus pulposus (NP), surrounded by the lamellae of the outer region known as the collagen-rich annulus fibrosus (AF) and sandwiched between the hyaline cartilaginous endplates (CEP) [18,19] (Fig. 1b). The IVD composite provides structural support and absorbs the shock of mechanical loading, while being the largest known avascular tissue in the human body with very limited intrinsic healing potential. Degenerative changes in the IVD are characterized by decreased proliferation and increased apoptosis of NP cells and their extracellular matrix (ECM) metabolism imbalance, dehydration and inflammation, aging of the AF, and calcification of the CEP. Nerves and blood vessels also grow into the disc tissue, especially in the inner regions of the AF and NP. These changes compromise the ability to maintain its normal hydrophilic properties, leading to significant water loss, partial atrophy of the NP, the disappearance of the normal boundary between the AF and the NP, and height loss of IVD, ultimately resulting in clinical symptoms [18,19] (Fig. 1c).

## 3 Biologic strategies in spinal fusion surgery

Due to anatomical variation and differences in surgical access, spinal fusion procedures differ significantly between the cervical and lumbar spine, particularly regarding the availability of autologous bone and the selection of biologic graft materials. In cervical fusion, commonly utilized techniques include posterior cervical fusion (PCF) and anterior cervical fusion approaches, the latter encompassing anterior cervical discectomy and fusion (ACDF) and anterior cervical corpectomy and fusion (ACCF) (Fig. 1d–e). ACDF accesses the IVD *via* an anterior approach and allows for limited harvesting of local autologous bone, such as osteophytes or bone from the anterior vertebral margin. However, the quantity is typically insufficient, necessitating supplementation with iliac crest bone grafts (ICBGs), allograft bone, demineralized bone matrix (DBM), or synthetic bone substitutes, which are often packed into an interbody fusion cage. ACCF, which involves the removal of one or more vertebral bodies and adjacent IVDs, is typically indicated for multilevel pathology or vertebral body fractures associated with spinal cord compression. While a greater volume of autologous bone may be harvested during ACCF, structural reconstruction typically relies on large allograft bone blocks, titanium mesh, or demineralized bone fiber (DBF), in combination with anterior plate-screw constructs for stabilization. PCF utilizes a posterior approach to expose the lamina and facet joints, enabling the harvest of limited autologous bone from the spinous processes and laminae. However, in multilevel fusion cases, the amount of local bone is often insufficient, and supplementary grafting with ICBGs, allograft, or synthetic bone substitutes is commonly required.

In the lumbar spine, fusion techniques are generally classified based on the site of bone graft placement: posterolateral lumbar fusion (PLF) or lumbar interbody fusion (LIF). PLF promotes arthrodesis by placing bone grafts between the transverse processes [20], typically relying on autologous bone harvested from decompression procedures, such as the spinous process, lamina, and facets. However, in multi-level or osteoporotic cases, the quantity of local bone is often insufficient, and additional graft materials, such as ICBGs, allografts, DBM, or synthetic bone substitutes, are commonly used to enhance fusion. In contrast, LIF involves the insertion of an interbody implant (e.g., cage, spacer, or structural graft) into the disc space following disc removal and endplate preparation [21]. LIF can be performed *via* several approaches, including posterior lumbar interbody fusion (PLIF), transforaminal lumbar interbody fusion (TLIF), oblique lumbar interbody fusion (OLIF), anterior lumbar interbody fusion (ALIF), and lateral or extreme lateral interbody fusion (LLIF or XLIF) (Fig. 1f–g) [21]. PLIF enables access to the disc space through medial facetectomy and dural retraction, allowing comprehensive disc and endplate preparation. Abundant autologous bone can be harvested from the lamina, spinous process, and articular processes, which can be used within interbody devices or along the fusion bed. TLIF, performed *via* a unilateral transforaminal approach, minimizes neural retraction while still permitting adequate disc space preparation and harvest of local bone. Fusion can be enhanced by adjunctive use of DBM, allograft, or synthetic bone grafts. OLIF utilizes a minimally invasive retroperitoneal corridor, accessing the disc space anterior to the psoas major muscle. ALIF involves an anterior retroperitoneal approach that exposes the entire ventral surface of the vertebral endplates, permitting the placement of large interbody devices or structural bone blocks. LLIF (or XLIF) accesses the disc space through the lateral retroperitoneal, transpsoas corridor. In OLIF, ALIF, and LLIF procedures, the posterior spinal elements are not involved, thus, local autologous bone cannot be harvested intraoperatively. Consequently, these approaches rely entirely on exogenous graft materials such as ICBGs, allograft, DBM, or synthetic substitutes to facilitate fusion.

## 4 Biologics in spine fusion surgery

### 4.1 Updated bone reconstruction algorithm

For the bone reconstruction algorithm, four different levels are currently commonly used, including allograft, bone substitute, autograft, and vascularized bone flap (VBF) [27]. With the recent introduction of several vascularized bone grafts (VBGs) for spinal fusion and bone reconstruction, the algorithm was proposed and expanded to six separate levels: allograft, bone substitute, nonvascularized bone graft (N-VBG), VBG, pedicled VBF, and free VBF [27]. To clarify the previously ambiguous and confusing terminology, the terms "flap" and "graft" have recently been distinctly defined [27]. "Graft" refers to tissue transplanted without a blood supply, relying on the vascular network of the recipient site for survival or integration [27]. "N-VBG" encompasses traditional cortical, cancellous, and corticocancellous grafts. “VBG” refers to the graft blood supply provided directly by muscle attachments and unnamed small periosteal feeders. "Flap" refers to tissue that maintains its blood supply when transplanted [27]. “Pedicled VBF” describes a grafted bone flap functionally supported by pedicled muscle tissue without the need for vascular anastomoses. “Free VBF” refers to a grafted bone flap transplanted along with its vascular supply (both arterial and venous) and requiring vascular anastomoses.

### 4.2 Autografts

Autografts are transplanted bone tissue derived from the host itself, including N-VBG and VBG, according to the updated bone reconstruction algorithm [27]. The two most common grafts used in spinal surgery are local autografts and ICBGs [28]. Autogenous bone has three essential properties (including osteoconduction, osteoinduction, and osteogenesis) required for bone formation, and does not carry associated immune or infection-related risks [[28], [29], [30]]. Available autografts include bone marrow grafts, non-vascularized cortical, and vascularized cortical and cancellous [30]. Cancellous bone promotes angiogenesis, cell recruitment, and osteoid deposition and mineralization, while cortical bone provides higher stability but has relatively lower vascularization and bioactivity [28,30]. In this investigation, autografts were categorized into two groups based on the necessity of a separate incision to obtain bone tissue: separate incision autografts (including the ICBGs, clavicle, manubrium sternum, and fibula) and local autografts (including facet joints, lamina, and spinous process, rid).

#### 4.2.1 Separate incision autografts

ICBGs are considered the "gold standard" for bone grafting in spinal fusion surgery, which provides both cortical and cancellous bone for bony fusion and mechanical support [28,31,32]. Cho et al. recently found racial differences in the cancellous bone composition of ICBGs [33]. They can be obtained through a posterior or anterior approach along the iliac crest [32]. However, the volume of ICBGs is limited, and their harvest increases operation time [28]. Donor-site complications are common and have been reported in the past, including pain, infection, sensory abnormalities, hematomas, scarring, graft site fracture, superior gluteal artery injury, iatrogenic cluneal nerve and lateral femoral cutaneous nerve injuries, as well as herniation of tissue through the donor site [31,32]. Persistent postoperative pain at the donor site is a significant issue, with 21.1 % of patients continuing to have pain ≥2 years after surgery, and 9.9 % of patients requiring long-term analgesics to relieve graft site pain [34]. However, recent investigations have found that it may be an overestimate as technology improves over time, which also can be confounded by the original surgical site or residual back pain, as patients cannot accurately identify the side of the iliac crest for bone grafting [35,36]. Therefore, it is believed that donor site pain should not be the primary reason for using bone graft alternatives for lumbar spine fusion. Recent improvements have focused on innovations in harvesting techniques, such as hinge-roof reconstruction [37] and trephine technique [34], and donor-site defect reconstruction techniques [38,39]. Although ICBGs remain a highly effective option for spinal fusion, their use has declined over the past decade due to the complications associated with donor sites [40].

**Fig. 2:**
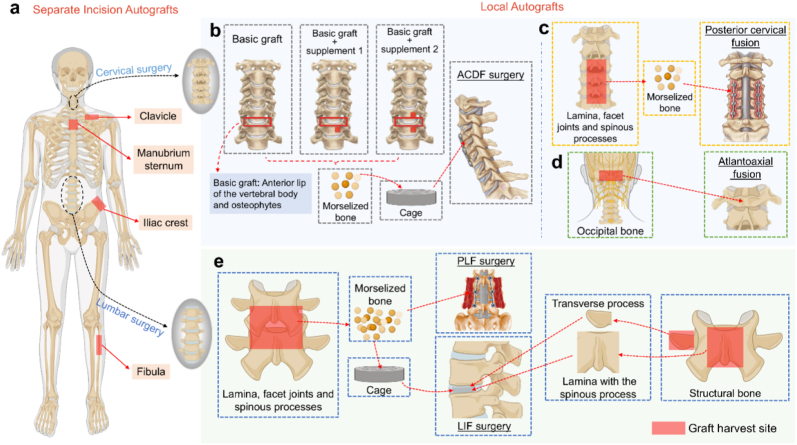
Current autograft sources for anterior and posterior cervical and lumbar fusion surgeries. **(a)**. Separate incision autografts used in spinal fusion surgery include the iliac crest, manubrium sternum, clavicle, and fibula. **(b).** Local autografts for anterior cervical discectomy and fusion (ACDF) typically involve bone grafts from the anterior lip of the vertebral body and osteophytes. These may be supplemented by partial vertebral resection if necessary, such as creating a 5 mm × 5 mm x 6 mm (depth) in the lower left quadrant of the vertebra (Supplement 1) [44] or a bony groove 2–3 mm from the upper and lower vertebrae of the surgical stage (Supplement 2) [55]. **(c).** For posterior cervical fusion (PCF), local autografts are commonly obtained from the facet joints, lamina, and spinous process of the surgical segment. **(d)**. Local autografts for atlantoaxial fusion surgery can be sourced from the occipital bone. **(e)**. In posterior lumbar fusion surgery, autografts are often taken from the facet joints, lamina, and spinous process, which are prepared into morselized bone for use in lumbar fusion procedures. These autografts can be applied directly in posterolateral fusion (PLF) or used to fill cages in lumbar interbody fusion (LIF) surgeries. Additionally, the transverse process or lamina with the spinous process can be used as structural bone for LIF surgery without a cage. The image was made on BioRender.com.

Other separate incision autografts reported for spinal fusion surgery include the clavicle [41], manubrium sternum [42], and fibula [43]. Each of these grafts carries specific risks and complications, such as a subclavian vessel or lung injury with clavicle grafting [44], reconstruction needs with manubrium sternum grafting [42], and potential donor site trauma with fibula grafting [43]. The fusion effect brought by these autografts is worth looking forward to, but the additional donor site trauma should also be considered (Fig. 2a).

#### 4.2.2 Local autografts

Local autografts refer to bone grafts that are actively or passively obtained directly from the surgical site without separate incisions. Common sources include the anterior lip of the vertebral body and osteophytes in anterior cervical spine surgery, and the facet joints, lamina, and spinous process of the surgical segment in posterior spinal surgery [45]. In addition, rids in the thoracic or thoracolumbar spine surgery and occipital bones in the atlantoaxial spine surgery are also applied. Local autografts may provide similar mechanical and physiological properties to ICBGs while avoiding donor-site complications [28,45]. Notably, the lamina and spinous process [46], and osteophytes are mainly composed of cortical bone [44].

Recent advancements in local grafts have primarily focused on the impact of volume and particle size on fusion and improvements in harvesting techniques. Quantification of local grafts shows that the lumbar spine provides more bone grafts than the thoracic spine, with the spinous processes contributing the most bone grafts during posterior spinal fusion in adolescent idiopathic scoliosis [47]. Recent research has also quantified the average volume and weight [48,49] of local grafts and confirmed their sufficiency for single-level and even for 3-level LLF [50]. In addition, the size of the local bone also affects the fusion effect [51,52]. Some researchers have demonstrated that local autografts can achieve equivalent results to structural ICBGs while causing less surgical trauma and shorter bone fusion times [53,54]. Innovations in local autografts include techniques for obtaining grafts from adjacent vertebral bodies during ACDF [44,55]. The above-mentioned local morselized bone can be categorized as N-VBG in the updated bone reconstruction algorithm [27]. Structural local autografts, such as the lamina with the spinous process [46,56] and transverse process struts [57,58], have recently been reported effectively used in thoracic and lumbar reconstruction and fusion surgeries for conditions like tuberculosis [46,[57], [58], [59]] and pyogenic spondylodiscitis [56,60].

Other local autografts, like occipital bones for atlantoaxial surgeries [61] and ribs for thoracic tuberculosis surgeries [62,63], have also shown promising results and can be classified as VBGs according to the updated BRCA [27]. The innovative use of local autografts in spinal fusion has garnered significant attention due to their mechanical support and fusion promotion, with ongoing research focused on optimizing harvesting methods and quantifying bone tissue availability (Fig. 2b–e).

### 4.3 Pedicled VBFs

**Fig. 3:**
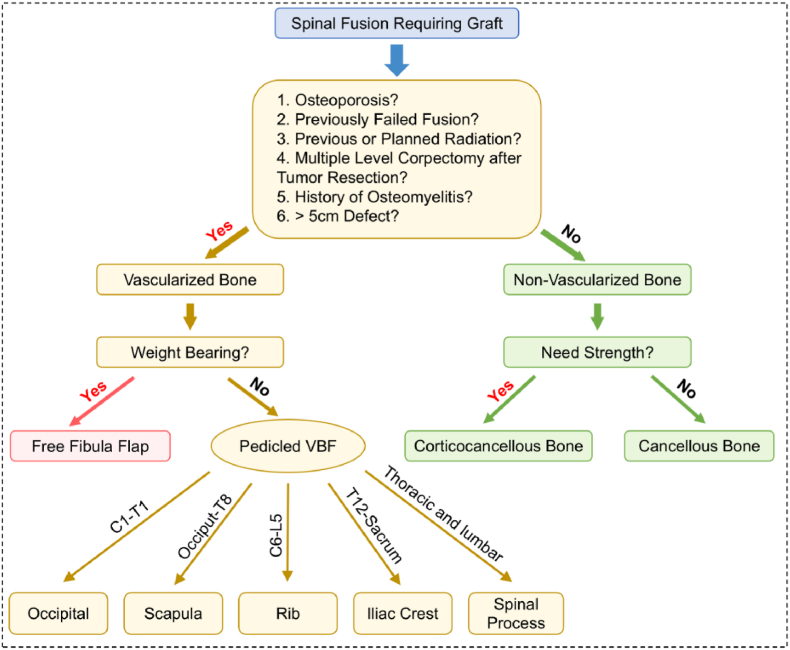
The decision tree for autograft selection in spinal surgery [65,69].

**Fig. 4:**
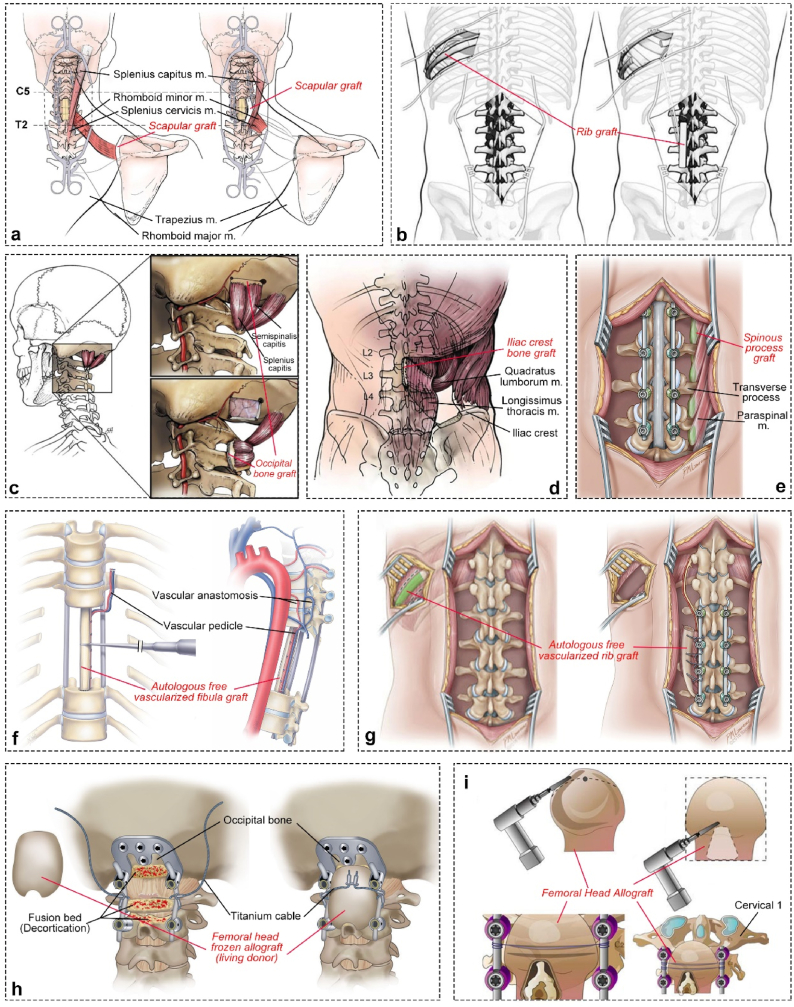
Schematic diagram illustrating the current use of bone grafts for augmented spinal fusion, including **(a**–**e)** pedicled vascularized bone flaps (VBFs), **(f**–**g)** free VBFs, and **(h**–**i)** femoral head allograft scaffolds. **(a**–**e).** Pedicled VBFs involve grafts from the pedicled **(a)** scapula [68], **(b)** rib [65], **(c)** occipital bone [66], **(d)** iliac crest [70], and **(e)** spinous process [71]. **(f**–**g)** Free VBFs are utilized in specific reconstructions, including **(f)** a free fibula VBF following tumor resection [76] and **(g)** a far lateral rib free VBF used in a cadaveric proof-of-concept study for spinal fusion [80]. **(h**–**i).** Schematic representations depict femoral head allograft scaffolds used for craniocervical fusion surgery [94,95].

Pedicled VBFs offer a regional option for transplanting vascularized bone which involves bone segments isolated and transported on their primary vascular pedicles [27]. As a recently developed bone grafting technique, pedicled VBFs address complex spinal fusion scenarios by providing a more accessible blood supply than traditional autografts, increasing osteoblast numbers, shortening arthrodesis time, offering biomechanical support during early bone healing, and achieving primary healing at the graft-host interface [64]. This avoids the progressive resorption, loss of structural integrity, pseudoarthrosis, and hardware failure observed in non-VBGs [64]. The decision tree to guide the selection of appropriate bone grafts is provided in Fig. 3 [[64], [65], [66], [67], [68], [69]]. Reported pedicled VBFs in spinal fusion surgery include the iliac crest [67,70], rib [65], lateral scapula [68], occipital bone [64,66], and spinal process [71]. Detailed techniques for each pedicled VBF procedure have been extensively summarized in recent articles (Iliac crest [67,72], rib [65,73], lateral scapula [68,74], occipital bone [64,75], and spinal process [71] (Fig. 4a–e).

### 4.4 Free VBFs

Free VBFs describe the transplantation of bone flaps with their vascular supply (arterial and venous) to the recipient site [27]. The free vascularized fibular flap is the most commonly used in spinal reconstruction. Recent work by Bongers et al. showed its effectiveness in spine reconstruction, particularly for the cervicothoracic spine, but the failure rate of lumbar implants remains high due to insufficient mechanical support [76] (Fig. 4f). Case reports have also documented the successful use of free vascularized fibular flaps after internal fixation failure in upper cervical spine reconstruction [77] and multiple failed occipitocervical fixation techniques [78]. While nonunion is a common complication, except for donor site complications, the risk is significantly reduced compared to nonvascularized fibular grafts [79]. In addition to the fibula flap, a recent cadaveric study has explored the feasibility of rotating far lateral rib grafts on the intercostal pedicle for augmented arthrodesis in the cervical or lumbar spine [80] (Fig. 4g). These innovative approaches highlight the potential of free VBFs in improving spinal fusion outcomes in challenging situations.

### 4.5 Allografts

Allografts involve the transplantation of bone from individual to another [31]. They are usually sourced from cadaveric or living donors when autografts are not available [31]. Compared with autologous bone, allografts are easier to obtain without donor-site complications for patients during the harvesting process [28,31]. Depending on the preparation methods, allograft bone can be divided into fresh, frozen and freeze-dried mineralized allografts, as well as DBM prepared by acid extraction of natural minerals [81]. Traditionally, allograft bone is available in various forms, including DBM, cortico-cancellous, morselized and cancellous chips, and osteochondral and whole bone segments [81]. Generally, allografts are primarily osteoconductive, have minimal osteoinductive potential, and are not osteogenic because the donor cells are eliminated during processing [81]. In addition, allogeneic bone has some disadvantages, such as slow integration with the host bone, lack of vascularization, and the risk of disease transmission and stimulation of the host immune response [28,31,82,83].

#### 4.5.1 Acellular mineralized allografts

Traditional allografts are usually acellular, having only osteoconductivity and weak osteoinductive properties. Recently, the research progress of acellular allografts mainly comes from the development of commercial products, while clinical research focuses on investigating the integration effects and complications caused by their application in various forms and surgical scenarios. Acellular structural allografts are used as intervertebral spacers for fusion or as intervertebral support in reconstruction surgery. Some studies showed that structural allografts provide better bone fusion than polyetheretherketone (PEEK) grafts [84] and have lower rates of pseudoarthrosis and reoperation [85] in overall spinal fusion surgery. Allografts in freeze-dried or fresh-frozen form used as intervertebral spacers have comparable healing rates in ACDF surgery [86]. Tricortical iliac crest allografts achieve good results in spinal tuberculosis, but the fusion time is longer than with autologous transplants [87]. Freeze-dried allografts, with or without autografts, can also be used as a treatment option for spinal fusion in spondylodiscitis [88]. Acellular non-structural allografts are often used as a filler graft for spinal fusion, with good results reported in combination with titanium mesh for spinal tuberculosis [89], or as a bone graft for the iliac crest graft donor site reconstruction [39]. However, caution is needed in athletes, as allografts may delay and reduce return to play [90]. Recent studies suggest that allografts can reduce transfusion rates, operation times [91], and hospital stays [92] compared to autografts. They are known to have a potential risk of infection that needs to be managed during procurement and sterilization [81], while recent investigations have shown that intraoperative swab culture results cannot predict this risk [93]. As surgeons closely collaborate with experts in tissue engineering, allografts can provide personalized, customized options for clinical applications. For instance, customizable femoral head allografts can serve as bone fusion scaffolds in rigid craniocervical fixation procedures [94,95] (Fig. 4h–i). This demonstrates an innovative application of allografts in specific scenarios to address the complexity of spinal anatomy.

#### 4.5.2 Cellular mineralized allografts

To address the lack of osteogenic properties of traditional mineralized allografts, viable cellular allografts or cellular bone matrices (CBMs) have been developed that are made from osteoconductive cadaveric bone with allogeneic stem cells [i.e., mesenchymal stem cells (MSCs)] retained or added to initiate osteogenesis [81]. MSCs can be isolated from various sources including bone marrow, placenta, umbilical cord blood, and more [81]. Given the properties of MSCs, specifically their ability to promote osteogenesis and evade the immune system, it is reasonable to assume that they could have a beneficial effect on spinal fusion [81].

Recent advances in CBMs primarily come from the development of commercial products, with clinical and preclinical studies focusing on their effectiveness in spinal surgery. Some clinical studies show promise in promoting cervical [96] and lumbar fusion [[97], [98], [99]], while preclinical studies demonstrate that cellular components of CBMs yield no additional benefit in spinal fusion [100], and the ability of different CBMs to induce successful fusion also varies widely [101]. A randomized clinical trial showed that the autologous MSCs + allograft group achieved higher rates of PLF at 6 and 12 months postoperatively compared to ICBGs [102]. However, MSC preparation in commercialized products is not standardized, and variations in donor age, donor site, and stem cell viability can affect the effectiveness of CBMs [81]. Recently, several reviews suggest that the potential of MSCs for promoting osteogenesis and spinal fusion has not been fully realized [81,[103], [104], [105]], because of MSC death caused by cell ischemia post-transplantation [103]. In addition, the high cost of CBM products, 4–5 times more expensive than DBM products and comparable in cost to recombinant human bone morphogenetic protein-2 (rhBMP-2), currently makes their use unjustifiable [103]. Therefore, the conversion of the osteogenic potential of MSCs pre-implantation into osteogenic effect *in vivo* is a direction that requires continued exploration by biologists and material scientists.

#### 4.5.3 DBM and DBF

DBM is typically made from cortical bone allografts that are cleaned and ground into particles, and treated with dilute hydrochloric acid to remove the mineral components, leaving a collagen matrix and other proteins [106]. It primarily consists of collagen (mostly type I, with some types IV and X), non-collagenous proteins, osteoinductive growth factors (e.g., BMPs), varying percentages of residual calcium phosphate minerals (1–6 %), and minor cellular debris [107]. DBM is osteoconductive and osteoinductive, with advantages such as no limitation on transplant volume, lack of donor-site complications, and shortened surgery and recovery time [107]. However, handling DBM can be challenging due to its various product forms, including powder or granules with a loose structure that may not stay firmly in the filling site and can be easily dispersed by flushing and blood flow during surgery, as well as prefabricated materials for filling with potential dead spaces [107]. Therefore, moldable bone paste or bone putty forms of DBM, which can be uniformly filled into the defect site and remain firmly, are the most popular [107]. Moldable DBM products are made of a composite material of DBM powder/granules and a biocompatible viscous carrier, which provides a stable suspension for the DBM powder or granules [107]. Cohesive carriers used for DBM can be divided into high molecular weight (e.g., collagen, chitosan, hyaluronic acid, carboxymethyl cellulose, and poloxamer 407) and low molecular weight materials [e.g., glycerol, calcium sulfate (CaS), bioactive glass (BG)] [107].

Recent advances based on DBM have primarily arisen from the development of commercial products and investigations targeting the effects of their clinical applications. Justin et al. have recently summarized the currently commercially available DBM-based products [81]. Commercial DBM putty products often consist largely of carriers (up to about 85 % carrier and 15 % active DBM), reducing the amount of active DBM components [108]. Carrier-combined DBM is not more effective than pure DBM as a bone graft augment, thus not supporting its combination with carriers to enhance spinal fusion [109]. DBM has been shown to be useful in lumbar fusion as a local autograft extender rather than a graft enhancer [106,110]. However, its properties depend largely on the preparation method, including demineralization processing technique, sterilization method, storage conditions, and donor age [106], which may explain inconsistencies between preclinical and clinical data on its osteoinductive potential [106]. The ability to easily deliver putty products to the implant site improves handling, but does not solve the problems of graft migration and compression resistance [106]. To address these challenges, DBF was developed, consisting of a coherent mass of elongated, mechanically entangled demineralized bone [111]. It can be formed into putties, strips, or other shapes, which improves handling, avoids graft migration, and provides resistance to compression, while maintaining similar osteoconductive and superior osteoconductive properties compared to DBM [106,112]. Market trends have shifted towards DBF-based products due to the perception that excipients dilute the active DBM content [106].

Regarding their effectiveness in spinal surgery, a meta-analysis showed that DBM combined with autografts for PLF and LIF had a slightly higher fusion rate than autograft alone, but not statistically significant [110]. Variability in fusion rates with DBM or DBF for spinal fusion has been observed across different studies [112], consistent with preclinical and clinical studies published before [113]. Notably, older age and lower bone density are risk factors for nonunion with DBM, which should be considered by surgeons [106]. The cost of DBM, though lower than that of rhBMP-2, remains high [114]. Despite the widespread use of DBM and DBF, high-quality evidence regarding their comparative effectiveness in spinal fusion is limited [106,112]. Optimizing the delivery of DBM through the development of new carriers has also attracted the interest of scientists. Several preclinical studies have investigated innovative carriers, such as synthetic alginate hydrogel carriers [115] and 3D-printed hydroxyapatite (HA) carriers [116,117]. The HA-DBM composite material induces a lower host inflammatory response than rhBMP-2 during bony fusion [118]. Looking ahead, combining DBM with other bone-promoting substances, such as autologous bone marrow MSCs, seed cells, growth factors, and so on, is considered a promising research direction [107]. This approach was recently highlighted by Roh et al., who demonstrated that adding Escherichia coli-derived rhBMP-2 into DBM contributes to improved fusion rates in a preclinical study [119].

### 4.6 Xenografts

Xenogeneic bone grafts involve bone tissue transplantation across species, commonly using bovine and porcine products [120,121]. Although xenografts offer a large supply and are low cost [121], they have high nonunion rates, risk of disease transmission, immune response of host tissue, and lack of living cells and osteogenic properties [120]. Clinical trials show mixed and controversial results, and current literature does not support their widespread clinical use [121]. Only one recent study using bovine-derived xenograft for spinal fusion in adolescent idiopathic scoliosis patients reported successful bone fusion at 24 months of follow-up [122]. Overall, xenografts have fallen out of favor in spinal surgery for both basic research and clinical applications.

### 4.7 Bone substitutes

#### 4.7.1 Synthetics

##### 4.7.1.1 Bioceramics

Bioceramics have gained prominence in bone tissue engineering due to their stable physical and chemical properties, excellent biocompatibility, osteoconductivity, and ability to promote biomineralization, while are also limited by drawbacks such as low elasticity, poor toughness, high stiffness, and brittleness [123]. They can be categorized into three types based on their interaction with living tissues post-implantation: 1) bioinert ceramics, such as zirconia and alumina, 2) bioactive ceramics, such as glass ceramics, BG, and HA, and 3) bioabsorbable ceramics, such as CaS, calcium silicate (CaSi), calcium phosphate (CaP) and calcium carbonate (CaC) [123].

Recent advances in clinical research on bioceramics have focused primarily on exploring their efficacy as bone graft extenders or intervertebral spacers in various spinal fusion surgeries. HA is the most important inorganic component of bone, and synthetic HA ceramics are often used as bone substitutes in spinal surgery due to their crystallographic and chemical similarity to human bone tissue [124]. Litak et al. summarized the use of HA products and their effectiveness in spinal surgery, particularly in applications such as intervertebral implants in ACDF, bone grafts in PLF, and pedicle screw coatings, all of which demonstrated good results [124]. Zhang et al. systematically analyzed nano-HA and its composites as grafts for intervertebral fusion similar to non-nano-HA grafts in terms of safety and efficacy in spinal reconstruction [125]. A meta-analysis by Liu et al. demonstrated that HA and its composites are comparable to autografts for clinical use in spinal reconstruction, with satisfactory efficacy and safety [126]. Yoshii et al. used HA/collagen composite loaded with bone marrow aspirate to achieve a similar fusion effect to that of local autografts in PLIF surgery [127]. BG is a bioceramic composed of silicon dioxide, sodium oxide, phosphorus and calcium oxide, which can produce different types of products by changing the ratio of components, with 45S5-bioglass being widely utilized for bone replacement [128,129]. Barrey et al. utilized 45S5 BG with local autografts (volume ratio: 1:1) in posterior spinal fusion surgery, yielding satisfactory outcomes in safety and bone fusion efficiency [130]. Westerlund et al. employed BG putty (BioSphere) with cancellous bone allograft (in ACDF and ALIF) or autograft (in TLIF) and achieved good results in terms of fusion rate (100 %) and clinical success rate (cervical spine is 93 % and lumbar spine reached 89 %) [131]. Compared with slowly degradable HA and BG, β-tricalcium phosphate (β-TCP) is a resorbable bioceramic with a solubility close to that of bone mineral, which does not dissolve under physiological conditions, but will be absorbed by cells (usually osteoclasts), and has osteoconductive and osteoinductive potential [132]. Recent studies have shown that porous *β*-TCP (Affinos®) achieved comparable intra-cage bone fusion rates to autografts in LLIF (70.9 % vs 58.6 %) [133]. Silicate-substituted calcium phosphate (SiCaP) is a bone substitute where some phosphate groups (PO_4_^3−^) are replaced by silicates (SiO_4_^4−^) in synthetic CaP, improving the osteoinductive properties [134]. A prospective, open-label study by Bolger et al. found that SiCaP-enhanced porosity (Inductigraft™, Altapore) improved clinical outcomes with a high rate of spinal fusion success (12 months: 86.3 %) in PLF surgery [135]. A recent systematic review by Cottrill et al. found no difference in fusion rates between patients treated with SiCaP and those who received rhBMP-2 substitute [134].

In addition, recent advances also include the development of new bioceramic-based composites or the utilization of bioceramic-based scaffolds to deliver drugs or bioactive factors, validating these approaches in preclinical spinal fusion models (Table 1). These innovations aim to provide more options for spinal fusion surgery. The delivery of rhBMP-2 is discussed in the separate rhBMP-2 section.

**Table 1:** Summary of recent studies in preclinical investigations of the use of bioceramic-based bone substitutes for spinal fusion.

Reference	Bone Substitutes	Application	Evaluation
Lu et al. [136]	Collagen/β-TCP composites (weight ratio: 4:1)	Posterior fusion	Rabbit
Ding et al. [137]	Collagen-HA with BMNCs^a^	PLF	Goat
Guo et al. [138]	Citrate-based tannin-HA composites	Posterior fusion	Rabbit
Östman et al. [139]	Calcium pyrophosphate (Ca-PP) with β-TCP	ACDF	Porcine
Daldal et al. [140]	Carbon nanotubes with HA-TCP composites	PLF	Rat
Elianna et al. [141]	Bioactive glass	PLF	Rat
Lin et al. [142]	PLGA/β-TCP composite + Salvianolic acid B	PLF	Rat
Conway et al. [143]	Nano-synthetic siliconized CaP putty	PLF	Rabbit
Salamanna et al. [144]	Sr-β-TCP^b^ + BMSCs	OP^c^ and non-OP PLF	Rat
Kwon et al. [145]	Whitlockite	PLF	Rat

a BMNC: bone marrow nuclear cells.

b Sr-β-TCP: Strontium substituted β-TCP.

c OP: Osteoporosis. PLGA: poly L-lactide-co-glycolic acid.

##### 4.7.1.2 Polymer

Recently, a conceptual experiment using a 3D foam of polycaprolactone (PCL) polymer doped with polymethacrylic acid and polydopamine achieved immediate stabilization of bone tissue and formed a trabecular-like structure, offering a new direction for interbody fusion without instruments [146]. Belluomo et al. reported that a novel polyethylene glycol/polylactic acid triblock copolymer binder provided cohesion, plasticity, and washout resistance during surgery. This adhesive was prepared into a ready-to-use formulation with biphasic CaP and then combined with autograft (volume ratio: 1:1) to achieve a comparable fusion rate with autograft in rabbit PLF surgery [147]. However, these findings indicate that polymers are not directly used to promote new bone formation and spinal fusion but can serve as adhesives to provide biomechanical support or as molding agents in biologics preparations to assist spinal fusion.

#### 4.7.2 Bioactive factors

##### 4.7.2.1 BMPs

Bone morphogenetic proteins (BMPs) were first reported by Dr. Marshal R. Urist in 1965 [148]. Due to the limited yield of naturally extracted and purified BMPs, rhBMPs have been produced using human recombinant gene technology since the mid-1990s [149]. Over 20 BMPs with osteogenic functions have been identified, with rhBMP-2 and rhBMP-7 being the most studied [150]. rhBMP-2 was approved by the FDA in 2002 for use in spinal surgery in combination with a metal interbody fusion cage for patients with skeletally mature L4-S1 primary degenerative disc disease [150,151]. rhBMP-7, also known as osteogenic protein-1 (OP-1), was approved by the FDA in 2001 [151]. However, both rhBMP-2 and rhBMP-7 have faced challenges such as high costs and safety issues (e.g., vertebral osteolysis, heterotopic bone formation, radiculitis, soft tissue swelling) [152]. Consequently, rhBMP-7 was withdrawn from the market in 2014 due to safety issues and financial controversies, and restrictions were imposed on the clinical application of rhBMP-2 [152]. Despite being FDA-approved only for single-level ALIF and OLIF at the L2-S1 level, rhBMP-2 is widely used (approximately 85 %) off-label in clinical [153]. A multicenter survey in France from 2011 to 2012 showed that rhBMP-2 was most commonly used in PLF, followed by ALIF, PLIF, LLIF, and TLIF [154]. In an updated review of the available evidence, Malham et al. summarized the recommended indications for the use of rhBMP-2 in spinal surgery [150] (Table 2). Some studies suggested that a clear informed consent process should be conducted between surgeons and patients regarding the current evidence regarding the benefits and risks of using rhBMP-2 and available alternative bone graft substitutes, both off-label and on-label [150,153], otherwise, the surgeon may be exposed to increased medical malpractice charges [153].

**Table 2:** Recommendations for rhBMP-2 use in different spinal surgeries [150].

Indication	Recommendation for rhBMP-2
Adult spine deformity	+++
Revision for pseudoarthrosis	+++
Lumbar fusion for degenerative disease	+++
Low-quantity or low-quality ICBG	+++
Posterior cervical fusion	++
Trauma	++
Tumor	++
Infection	++
ACDF	–

Notes: +++, excellent option; ++, good option; −, poor option or unsuitable. rhBMP-2, recombinant human bone morphogenic protein-2; ICBG, iliac crest bone graft; ACDF, anterior cervical discectomy and fusion.

Recent clinical studies continue to focus on the outcomes and complications of using rhBMP-2 in various spinal fusion procedures. In ACDF surgery, an exploratory meta-analysis by Wen et al. found rhBMP-2 has achieved a higher fusion rate but a higher complication rate, more dysphagia and wound infection compared with non-rhBMP-2 patients, and the recommended dose preferably <0.7 mg/level [155]. Similarly, Medina et al. reported a 100 % fusion rate with rhBMP-2 in ACDF, but dysphagia complications occurred [156]. In PCF surgery, Weinberg et al. reported that rhBMP-2 was not associated with a higher rate of early complications when the dose was minimized [157]. Overall, a meta-analysis conducted by Liu et al. reported that the use of rhBMP-2 demonstrated a higher fusion success rate and reduced the risk of reoperation, with no significant difference in complication rates compared to ICBGs in lumbar fusion [158]. In PLIF surgery, Trang et al. reported a lower incidence of nonunion and a significant improvement in efficacy, but transient radiculitis was common (42 %), whereas osteolysis and epidural cyst formation were rare [159]. Park et al. used HA/β-TCP hydrogel to deliver low-dose rhBMP-2, enhancing local autograft fusion rates in PLIF [160]. In LLIF surgery, Son et al. found that rhBMP-2 increased the fusion rate of DBM, while the effects of 1 or 2 mg rhBMP-2 per 5 mL of DBM paste were comparable [161]. In PLF surgery, meta-analytic investigations found that rhBMP-2 had a higher fusion rate, shorter operation time, lower blood loss, and fewer additional surgeries than ICBGs [162,163]. Furthermore, Choi et al. suggested that 2.5 mg rhBMP-2/segment was sufficient for PLF in a pilot study [164]. In spinal tumor surgery, Munim et al. reported that the use of rhBMP-2 in the USA from 2005 to 2020 significantly declined, with higher rates of surgical site and systemic infections, while the risks of mortality, implant failure, or reoperation were not significantly reduced [165].

In the field of preclinical studies, recent advances focus on optimizing the delivery of rhBMP-2 using various carriers, such as electrospun, synthetic bone void filler carrier composed of 40 % β-TCP, 30 % siloxane-containing vaterite, and 30 % poly L-lactide-co-glycolic acid (PLGA) [166], ECM and CaP infused with polydopamine carrier [167], and HA/β-TCP hydrogel carrier [168] to enhance the effect of spinal fusion. Our team recently used a CaS/HA ceramic carrier to deliver rhBMP-2 to achieve spinal fusion, and co-delivered zoledronic acid to inhibit the osteoclastogenic effect of rhBMP-2, significantly enhancing and accelerating spinal fusion and potentially reducing the dose of rhBMP-2 [169]. Beyond the carrier, Briquez et al. designed a bridge protein with a high affinity for rhBMP-2 and collagen, thereby optimizing the retention and efficacy of rhBMP-2 in spinal fusion [170].

In addition, BMP-6 also has a specific role in converting stem cells into cartilage- and bone-forming cells, being resistant to BMP antagonists that neutralize BMP-2 and having a 20-fold higher affinity for BMP receptor type IA (BMPR-IA) than BMP-7 [8]. Therefore, it is thought that much smaller amounts of rhBMP-6 are required for successful bone formation and healing. Recently, researchers from the University of Zagreb conducted a series of basic studies on rhBMP-6 for spinal fusion using autologous blood coagulum as a physiological matrix combined with allogeneic bone [171,172] or bioceramics [[173], [174], [175]] as a compressive matrix, achieving good results in rabbit and sheep models.

##### 4.7.2.2 ABM/P-15

ABM/P-15 is composed of an anorganic bovine-derived hydroxyapatite matrix (ABM) combined with a synthetic 15 amino acid sequence (P-15), which has both osteoconductive and osteoinductive properties [151,176]. ABM/P-15, marketed as i-Factor™ Bone Graft, has been FDA-approved since November 2015 for the reconstruction of degenerated IVDs from C3-4 to C6-7 following a single-level discectomy for refractory radiculopathy (arm pain and/or neurologic deficits) after failure of conservative treatment [151]. It must also be used within an allograft bone ring and in conjunction with anterior plate fixation. The initial U.S. FDA Investigational Device Exemption study produced similar results at 2 years post-surgery compared with local autografts [177].

Recent advances in ABM/P-15 research have reported long-term results in cervical surgery, demonstrating that ABM/P-15 (i-Factor) met all four FDA-mandated non-inferiority success criteria and confirmed its safety and efficacy up to six years post-surgery for single-level ACDF in the treatment of cervical radiculopathy [178]. Although preliminary results in cervical fusion are encouraging, full evaluation of its efficacy in lumbar fusion surgery is still in its infancy, and the use of ABM/P-15 in these procedures is currently considered “off-label” [179]. A randomized double-blind trial conducted by Jacobsen et al. showed that the fusion rate with 5 cc of ABM/P-15 bone putty plus local autografts was significantly higher than that with 30 g of allograft plus local autografts in non-instrumented PLF, but this did not translate into better clinical outcomes [180]. A retrospective observational study by Sathe et al. showed that ABM/P-15 seemed to achieve fusion earlier than rhBMP-2 and DBM in the treatment of degenerative lumbar spine disease with ALIF or OLIF plus pedicle screw fixation, while maintaining a similar clinical and complication profile [181]. A recent systematic review also presented that ABM/P-15 has a significantly faster fusion rate than traditional grafts, including allogeneic grafts, autologous grafts, DBM, and rhBMP-2 [179]. However, ABM/P15 appeared to exhibit the ability to migrate in the absence of external stabilization, resulting in a significantly lower fusion rate than the allograft group in a sheep PLF fusion model [176]. In conclusion, the current use of ABM/P-15, both labeled for cervical fusion surgery and off-label for lumbar fusion surgery, has shown positive results. However, as it has been on the market for a relatively short period, knowledge about its long-term efficacy and associated complications is still limited.

##### 4.7.2.3 PRP

Platelet-rich plasma (PRP) is obtained by centrifuging autologous peripheral blood and releases numerous growth factors, including transforming growth factor-β (TGF-β), platelet-derived growth factor (PDGF), fibroblast growth factor (FGF), vascular endothelial growth factor (VEGF) when activated [151,182]. Therefore, PRP is considered to be beneficial for spinal fusion surgery [182]. A recent clinical study using PRP combined with local autografts in PLF [183] and two preclinical studies using mineralized collagen carriers loaded with PRP [184,185] showed positive results. However, several recent systematic reviews demonstrated that PRP as a bioactive agent has limited efficacy in enhancing spinal fusion [[186], [187], [188], [189]]. Therefore, the effect of PRP in spinal fusion surgery requires further exploration, which may align with investigations into other cell-based products that are influenced by factors such as preparation technology and donor characteristics.

##### 4.7.2.4 rhPDGF-BB

Recombinant human platelet-derived growth factor B homodimer (rhPDGF-BB) is a growth factor approved by the FDA for surgical fusion of the ankle and/or hindfoot in 2015 [151]. Recently, it has been applied to spinal fusion surgery *via* utilizing β-TCP/collagen matrix delivery to promote spinal fusion in preclinical studies, making it a potential biological alternative [190,191]. Therefore, rhPDGF-BB might be a potential growth factor for spinal fusion, but its clinical outcomes and side effects need further investigation.

##### 4.7.2.5 Prostaglandin E2 receptor EP4 subtype agonists

Prostaglandin E2 (PGE2), a lipid mediator involved in inflammation and bone metabolism, regulates bone remodeling by acting through the EP4 [192] receptor. This mechanistic pathway has positioned selective EP4 receptor agonists as a novel and promising class of bioactive agents for enhancing spinal fusion. Leveraging this biological insight, our group recently developed KMN-159, a novel EP4 agonist that exhibited significant osteopromotive effects in a rat PLF model when delivered *via* a mineralized collagen matrix scaffold [193]. These preclinical results highlight the therapeutic potential of EP4 receptor agonists in spinal fusion applications. Despite encouraging early data, further investigation is required to assess the long-term efficacy, safety profile, and clinical translatability of EP4 agonists in human spinal fusion procedures.

## 5 Additive manufacturing (AM)

AM has revolutionized various industries by offering unprecedented flexibility, customization, and efficiency through building objects layer by layer from digital designs. Based on the International Organization of Standardization, there are 7 a.m. categories: vat-polymerization, material extrusion, material jetting, binder jetting, powder-based fusion, sheet lamination, and direct energy deposition [194]. Specific techniques within these categories include: stereolithography (SLA), direct light processing (DLP), liquid crystal display based (LCD), continuous liquid interface (CLIP), 3D (bio)printing, selective laser sintering (SLS), selective laser melting (SLM), electron beam melting (EBM), fused deposition modeling (FDM) and emerging technologies of volumetric additive manufacturing (VAM) and 3D nanoprinting. In addition, electrostatic spinning, although not traditionally classified under conventional AM, is still capable of preparing fiber scaffolds with a 3D structure, and in recent years, electrostatic spinning has also introduced the concept of AM, which enables more precise control of fiber deposition. Techniques in this area include solution electrospinning, melt electrospinning, near-field electrospinning and melt electrowriting [195,196].

**Fig. 5:**
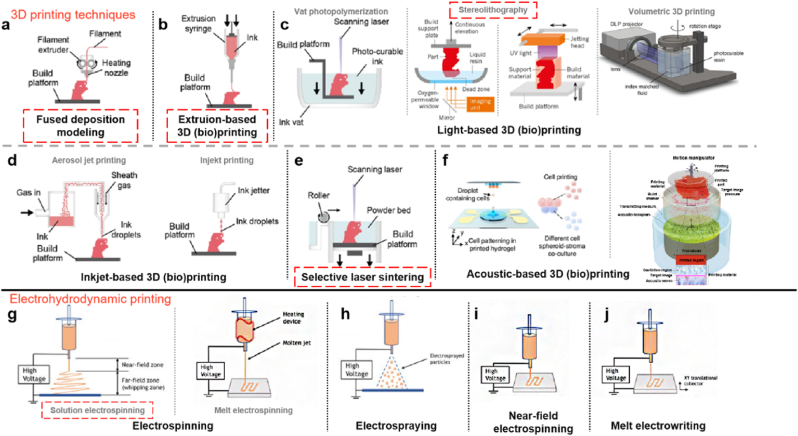
The schematic depiction of the principles behind the above-mentioned additive manufacturing (AM) technologies [195,[201], [202], [203], [204], [205]], and technologies currently used in the spinal surgery are marked with red boxes.(**a**) Fused deposition modeling; **(b)** Extrusion-based 3D (bio)printing; **(c)** Light-based 3D (bio)printing; **(d)** Inkjet-based 3D (bio)printing; (**e**) Selective laser sintering; (**f**) Acoustic-based 3D (bio)printing; (**g**) electrospinning; (**h**) Electrospraying; (**i**) Near-field electrospinning and (**j**) Melt electrowriting. (Technologies have been used in spinal surgery are marked with red dotted boxes). (For interpretation of the references to colour in this figure legend, the reader is referred to the Web version of this article.)

In spine surgery, AM offers significant advantages in creating complex geometries and customized implants tailored to individual patient anatomy, effectively addressing the intricate nature of spinal anatomy. This section focuses on the recent advances in crucial AM technologies within the context of spinal surgery, including 3D printing and electrospinning (Fig. 5).

### 5.1 D printing

3D printing has enabled personalized treatment strategies, enhanced surgical planning, and improved patient outcomes in spinal disorders. The current 3D (bio)printing technologies were summarized as shown in Fig. 5a–f [197]. Those applied in spinal tissue engineering are highlighted with red dashed boxes and further elaborated below.

**Extrusion-based printing:** It is the most commonly used 3D printing technique, like fused deposition modeling (FDM) (Fig. 5a) and extrusion-based 3D bioprinting, the objects are created by deposition of extruded materials through a nozzle strand-by-strand and layer-by-layer according to the generated controlling code of computer-aided design (CAD). These methods accommodate a variety of materials, including natural and synthetic materials with shear-thinning properties and additional components, to achieve the desired results (Fig. 5b).

**SLA:** which utilizes specific light sources, like UV light, to cure the liquid photopolymer resin layer by layer to build the 3D structure based on a digital 3D model making it a pioneering technology in rapid prototyping and manufacturing. The UV laser traces the cross-section of the object onto the surface of the liquid resin, solidifying it to form a thin layer (Fig. 5c).

**SLS:** The principle of SLS is using a high-powered laser to selectively melt and sinter powdered materials like thermoplastics or metals to create objects. The laser traces each cross-section on the powder bed, curing it layer by layer, with the bed being lowered and re-coated with powder until the object is complete (Fig. 5e).

### 5.2 Electrospinning

Electrospinning, as one of the representative electrohydrodynamic micro-to nanoscale fabrication techniques (Fig. 5g–j), has been extensively applied in tissue engineering and regenerative medicine due to its capacity to produce fibrous scaffolds that mimic the native extracellular matrix [196,198]. It is a simple, versatile, and controllable technique to produce micro- and nano-scaled fibrous structures *via* utilizing polymer solutions or melts under an electrostatic field. The typical device for electrospinning usually requires a syringe (containing the polymer solution) with a metal needle to conduct electricity, a syringe pump to regulate the flow rate, a high-voltage power supply, and a metal collector [196,199].

During electrospinning, the liquid is extruded from the spinneret and produces hanging droplets due to surface tension. When energized, electrostatic repulsion between surface charges of the same sign deforms the droplet into a Taylor cone from which a charged jet is ejected. The jet initially extends along a straight line and then undergoes a violent whipping motion due to bending instability. As the jet is stretched to finer diameters, it rapidly solidifies, leading to the deposition of solid fibers on a grounded collector [195,200] (Fig. 5g).

### 5.3 D printing applications in spinal surgery

#### 5.3.1 Anatomical models for preoperative planning and training

**Fig. 6:**
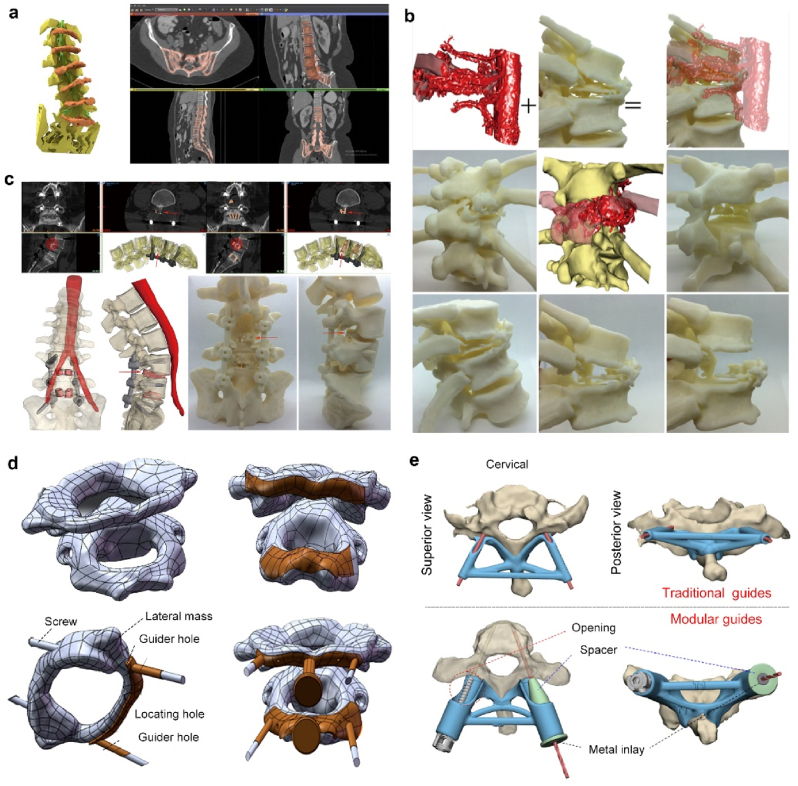
Examples of 3D printed **(a**–**c)** anatomical models and **(d**–**e)** screw guides in spinal surgery. **(a).** Screenshot of lumbar spine CT segmentation by computer (left) and the 3D printed spinal anatomical model (right) [206]. **(b**–**c).** Examples of 3D printed anatomical models for preoperative planning by **(b)** depicting the complex vascular distribution of spinal tumors or **(c)** complex revision surgeries (L4-S1) [208]. **(d).** Design of the guide template for posterior atlantoaxial pedicle screw implantation [214]. **(e**). Two types of pedicle screw implantation guides, including traditional personalized drilling guides (above) and innovative modular guides (below) [215].

3D printing has significantly impacted preoperative planning and surgical training by allowing the creation of anatomically accurate replicas of the spine. These physical models assist surgeons in simulating complex procedures, assessing surgical approaches, and anticipating potential challenges before entering the operating room. As a result, this preoperative rehearsal enhances surgical precision and reduces operative time and intraoperative complications. For example, Li et al. created a 3D model of a degenerative lumbosacral spine with scoliosis and spondylosis by 3D printing utilizing different materials to mimic bone and soft tissue (Fig. 6a) [206]. These models were used to train medical students without prior exposure to CT-guided spine surgery and helped novices gain the skills and confidence to perform such surgeries. Similarly, Coote et al. reported three cases of complex pediatric spinal surgery using 3D printed anatomical models, including congenital scoliosis and atlantoaxial instability, which helped surgeons to fully understand the anatomical structure of the patient's complex spinal deformity pre-operation, thereby better preparation for surgery, reducing operation time and minimizing surgical risks [207]. Parr et al. documented 3D printed models depicting the intricate vascularity of spinal tumors, facilitating the mitigation of inadvertent hemorrhagic events during surgical interventions, and the planning of complex revision surgeries for the removal and insertion of internal fixation devices [208] (Fig. 6b–c). In addition, 3D-printed spine models can also be used for scientific research, such as testing the performance of 3D-printed cages [209].

#### 5.3.2 D printing-based screw guides

Using 3D-printed screw guides in challenging spine surgeries can effectively reduce the risk of complications. Pedicle screws are the workhorse of modern spinal instrumentation but are associated with issues such as screw misalignment and pedicle wall perforation [[210], [211], [212]]. Pediatric cervical and thoracic fixation, pedicle sclerosis or deformation, and spinal deformities are particularly prone to pedicle screw misalignment. The application of 3D printed guides/templates for pedicle screw implantation is considered a cost-effective and promising adjunct. Dong et al. utilized 3D printing to create an anterior cervical pedicle screw guide, a lesion osteotomy guide, and a personalized anterior bilateral pedicle screw-fixed artificial vertebra for the treatment of cervical tuberculosis [213]. Wang et al. fabricated a 316L stainless steel posterior atlantoaxial pedicle screw guide using SLS to assist surgeons in inserting high-precision screws into the spine (Fig. 6d) [214]. In addition, Pijpker et al. used 3D printing to develop an innovative modular guide (drilling and screwing are integrated into a single device) based on the traditional drilling guide and showed that the modular guide significantly improved the accuracy of pedicle screw insertion in the cervical spine, while in the cervical spine, both guides were highly accurate (Fig. 6e) [215].

#### 5.3.3 D printing-based internal fixation implants

Currently, 3D-printed internal fixation implants in spinal surgery mainly include three categories: AVB, cage, and pedicle screw, of which the first two are more widely used. The fabrication and application of spinal surgery implants *via* utilizing different 3D printing techniques in recent years are summarized and listed in Table 3.

**Table 3:** Summary of recent advances in 3D printed spinal implants including AVBs, cages, and screws.

3D printing	Modality	Material	Application	Evaluation	Reference
SLS	AVB	Ti6Al4V powder	Thoracolumbar Reconstruction	Clinical	Zhou et al. [216]
			ACCF	Clinical	Fang et al. [217]
			ACCF	Rabbit	Hossain et al. [218]
			Lumbar Reconstruction	Goat	Zhang et al. [219]
	Cage	Ti6Al4V powder	Anterior Atlantoaxial Fixation and Fusion	Clinical	Wang et al. [220]
			LLIF	Clinical	Adl et al. [221]
				FEA^a^	Wu et al. [222]; Mehboob [223].
			XLIF	Ovine	Fogel et al. [224]
			XLIF	Rhesus Macaques	Zhang et al. [225]
				*In Vitro*^b^	Pan et al. [226]; Basgul et al. [227]
				Cell Experiment	Żak et al. [228]
	Screw	Ti6Al4V		FEA^a^	Yao et al. [229]
FDM	Cage	PEEK/Ta^c^	ACDF	Sheep	Jia et al. [230]
		Polylactide (PLA)	OLIF	Clinical	Shih et al. [209]
		PCL/β-TCP	PLIF	Clinical	Liu et al. [231]
		PCL	ACDF	Porcine	Ho et al. [232]
SLA	Vertebra	β-TCP powder		*In vitro*	Zhou et al. [233]

a FEA: finite element analysis; AVB: artificial vertebral body.

b *In Vitro* indicates only characterization, without cell-based and *in vivo* experiments.

c PEEK/Ta indicates the polyetheretherketone/tantalum composite material.

**Fig. 7:**
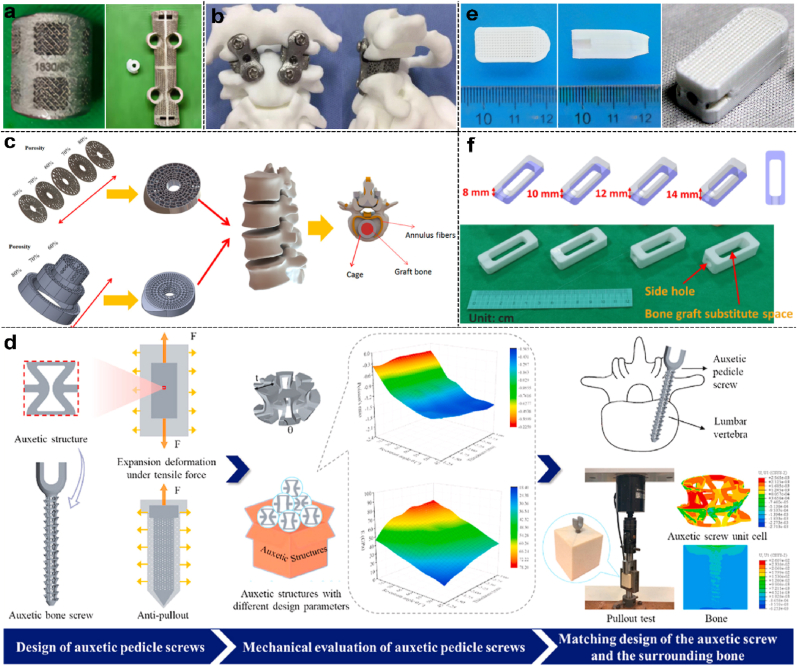
Application of 3D printing in implants for spinal surgery, including artificial vertebral bodies, cages, and screws. **(a).** The comparison images of an off-the-shelf (left) and a custom 3D-printed (right) artificial vertebral body for spinal reconstruction [216]. **(b)**. Schematic diagram of 3D printed locking cage for the treatment of atlantoaxial subluxation [220]. **(c)**. Schematic diagram of the structural optimization of 3D-printed titanium cages featuring gradient porosity [226]. **(d).** Design and mechanical testing of 3D-printed expandable titanium alloy pedicle screws [229]. **(e).** Visual appearance of a biodegradable cage fabricated by FDM with PCL/TCP biomaterials from different angles [231]. **(f)**. Visual appearance of biodegradable cages with side holes made by 3D printing with PLA biomaterials at various heights [209].

Titanium alloys, known for their high strength, biocompatibility, and corrosion resistance, are widely used to manufacture internal fixation implants. Due to its metallic nature, SLS technology is often employed to create AVBs, cages, and screws in spinal surgery. Compared with traditional AVB or titanium mesh, recent advancements include the development of AVB integrated with the fixation system with 3D printing for reconstruction after thoracolumbar tumor resection [216] (Fig. 7a) and ACCF surgery [218]. Another innovation involves manufacturing a movable lumbar vertebral complex, aimed at reconstructing the height and stability of the surgical segment while preserving its motion function [219]. Similarly, Wang et al. customized a titanium alloy locking cage, integrating the cage with the fixed steel plate for the treatment of atlantoaxial subluxation [220] (Fig. 7b). While titanium alloy intervertebral fusion cages offer numerous benefits, they also have certain disadvantages, such as being significantly stiffer than bone. This can lead to stress shielding, a phenomenon where the metal cage bears most of the load, thereby reducing the mechanical stimulus required for bone growth and remodeling and potentially leading to bone resorption or weakening around the implant [234]. To mitigate stress concentration caused by the high strength of titanium alloy, Pan et al. optimized and designed a cage with gradient porosity using SLS technique based on finite elements, which showed that the stress-strain distribution of the cage was consistent with that of the human skeleton [226] (Fig. 7c). In addition, Yao et al. recently used SLS technology to produce a tension-expandable pedicle screw that expands radially under tension to resist pullout and improve bone screw fixation. This innovation offers a potential customized design for bone-specific anti-pullout screws and provides new insights into addressing issues of screw loosening and pullout, especially for those with osteoporosis [229] (Fig. 7d).

In recent years, the application of biodegradable materials in spinal fusion devices has attracted extensive attention from researchers worldwide. Compared with titanium alloys, degradable material devices have less stress masking while still maintaining sufficient mechanical strength to ensure initial stability. Meanwhile, the devices will degrade gradually with new bone formation until fully degraded, thereby reducing the long-term complications. For instance, Liu et al. prepared a novel PCL/TCP biodegradable cage using FDM, demonstrating excellent support strength, histocompatibility, and osseointegration in clinical trials [231] (Fig. 7e). Jia et al. used degradable PEEK composited with tantalum metal to guide cellular behavior and promote new bone infiltration, resulting in accelerated osseointegration *in vivo* [230]. Shi et al. fabricated cages with side holes using PLA biomaterial and optimized the method for filling these cages with biologics to reduce the scattering during implantation and enhance the contact between the cage and the endplates [209] (Fig. 7f).

#### 5.3.4 D printing-based tissue regeneration scaffolds

With the widespread application of 3D printing in biomedical research and clinical settings, recent studies increasingly focus on developing tissue substitutes for repair and regeneration. In spinal research, the fabrication and preclinical investigation of bone and IVD-engineered scaffolds are central to current research efforts, and significant progress has been made in these areas.

##### 5.3.4.1 Bone regeneration scaffolds

**Fig. 8:**
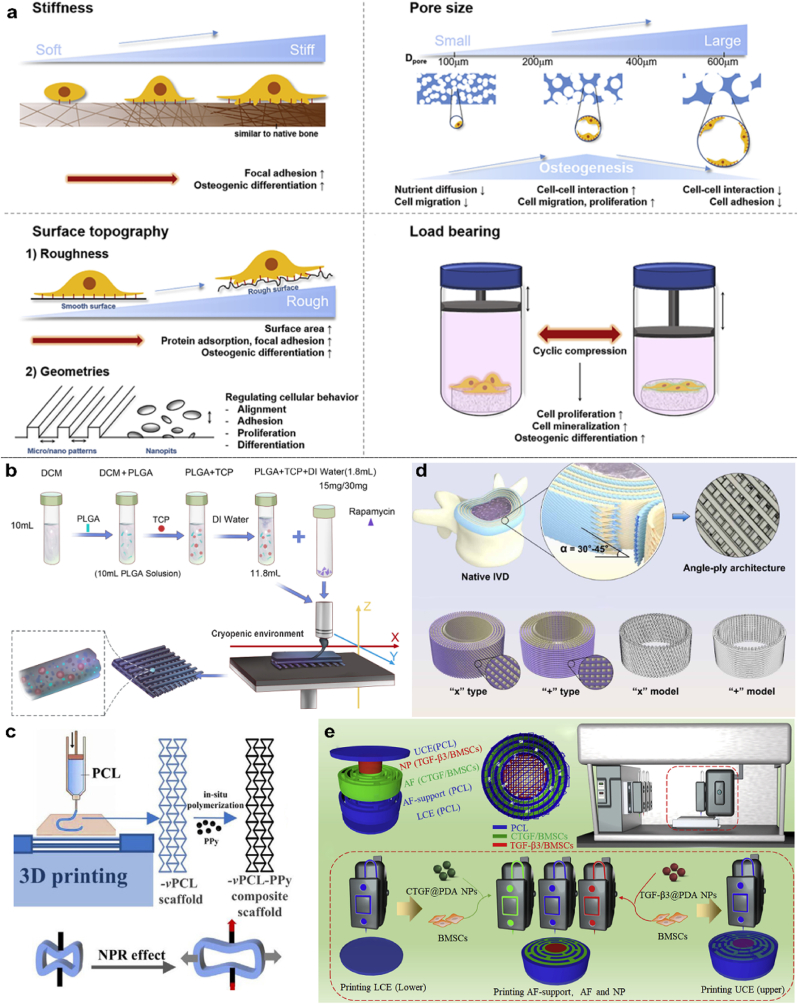
Application of 3D-printed tissue regeneration scaffolds in spinal surgery, including **(a)** scaffold design parameters in bone tissue engineering, **(b)** bone tissue engineering scaffolds, and **(c**–**e)** IVD tissue engineering scaffolds. **(a).** Schematic illustration of scaffold properties for bone-tissue engineering to enhance osteogenic differentiation [235]. **(b).** Schematic diagram of the process for 3D printing a bone tissue composite scaffold for spinal fusion (3D: three-dimensional; DCM: dichloromethane; DI: deionized; PLGA: poly (D, L-lactic-co-glycolic acid); TCP: β-tricalcium phosphate) [238]. **(c).** Schematic diagram of 3D-printed PCL scaffolds with a negative Poisson's ratio effect designed as AF scaffolds [241]. **(d).** Design of AF scaffolds created by 3D printing, featuring a biomimetic pore structure that mimics natural AF tissue, based on finite element analysis [244]. **(e).** IVD scaffolds incorporating dual growth factor (GF) release, fabricated using 3D bioprinting, and a PCL framework fabricated using 3D printing [247].

Bone is a structurally and biologically complex 3D tissue that serves both as a load-bearing framework and as a dynamic environment for cellular activities. Consequently, an ideal bone tissue regeneration scaffold must exhibit excellent biocompatibility while providing a well-balanced combination of mechanical strength, architectural design, and biological functionality [235]. To ensure mechanical compatibility with native bone, scaffolds should have an elastic modulus in the range of 0.1–2 GPa for cancellous bone and 15–20 GPa for cortical bone. Architecturally, interconnected pores of 200–400 μm are considered optimal to facilitate nutrient diffusion, vascular ingrowth, and osteogenesis. Additionally, micro-to nanoscale surface topography, typically ∼1–2 μm roughness or submicron features, has been shown to enhance cell adhesion, proliferation, and osteogenic differentiation. From a biomechanical perspective, scaffolds should also withstand physiological loading, as cyclic mechanical stimulation (∼10 % strain at 0.5–1 Hz) can significantly promote osteoblastic activity and ECM mineralization [235] (Fig. 8a). Recent preclinical studies have demonstrated the effectiveness of biologically and mechanically optimized 3D-printed scaffolds in promoting spinal fusion. For example, composite scaffolds combining HA and DBM at a 3:1 HA:DBM ratio achieved the highest fusion rates, significantly outperforming DBM-only and other HA:DBM formulations (1:1, 1:3) [236]. When fabricated with 45° aligned struts and 1000 μm macropores, these scaffolds exhibited superior bone ingrowth and full vascularization [237], along with reduced inflammatory responses compared to rhBMP-2-treated controls [118]. Similarly, β-TCP/PLGA composite scaffolds loaded with 15–30 mg of rapamycin have been shown to enhance lumbar fusion by regulating the *in-situ* activity of osteoblasts and osteoclasts without requiring exogenous growth factors [238] (Fig. 8b). In terms of mechanical performance, PEEK/silicon nitride scaffolds with a triply periodic minimal surface (TPMS) structure and 30 % porosity achieved a compressive strength closely matching that of trabecular bone and exceeding the mechanical performance of higher-porosity designs (50 % and 70 %) [239]. Overall, these findings underscore the critical role of microstructural and compositional optimization in the development of next-generation bone regeneration scaffolds. However, most current approaches still emphasize structural regularity over personalized-specific geometries, which remains a major challenge for clinical translation.

##### 5.3.4.2 IVD regeneration scaffolds

IVD scaffolds are bioengineered structures that mimic the natural properties of IVDs to support and promote tissue regeneration. They are designed to replace or repair damaged IVDs, restore their functions, provide structural support, maintain disc height, and enable the absorption of mechanical loads. Based on their simulated structure and function, current IVD scaffolds mainly include AF scaffolds [[240], [241], [242], [243]] and whole IVD (AF-NP) scaffolds [[244], [245], [246], [247]]. Christiani et al. created PCL scaffolds using FDM as an AF scaffold, results showed that the scaffold contributes to promoting AF regeneration [240]. Jiang et al. 3D printed PCL scaffolds with negative Poisson's ratio effect and polypyrrole coating, which can withstand spinal axial loads and resist NP swelling while showing uniform stress diffusion under NP expansion and contraction, thus can be used as AF scaffolds [241] (Fig. 8c). Costa et al. utilized enzyme-crosslinked silk fibroin/elastin bioink [242], and Marshall et al. employed flexible PLA [243] to print AF scaffolds, both demonstrated potential as engineered IVD scaffolds. Gloria et al. fabricated AF-NP-engineered scaffolds by printing PCL scaffolds filled with collagen-LMW HA-4S-StarPEG-CNPs hydrogel loaded with human MSCs [245]. Liu et al. used 3D-printed PCL scaffolds filled with GelMA hydrogel to obtain engineered AF-NP scaffolds, demonstrating their potential in a rat total IVD replacement model [244] (Fig. 8d). Wu et al. 3D printed a PLA framework and then filled it with MSC cell-laden poly (ethylene glycol) diacrylate (PEGDA) by bioprinting as an engineered IVD tissue scaffold that could maintain disc height and promote the deposition of proteoglycan and collagen in a rat total IVD replacement model [246]. Sun et al. utilized 3D bioprinting technology to mix bone marrow MSCs (BMSCs) and TGF-β3 with hydrogel to print the scaffold core, while BMSCs and CTGF were combined with hydrogel to print the surrounding structure of the scaffold. TGF-β3 and CTGF induced the differentiation of BMSCs into NP cells and AF cells, respectively, to replicate the biological function of IVD tissue. Additionally, the bioprinted scaffold was integrated with a printed PCL framework to provide mechanical support for the entire IVD scaffold [247] (Fig. 8e).

### 5.4 Electrospinning applications in spinal surgery

One of the main advantages of electrospinning is the ability to generate 3D scaffolds with tailored structural features to mimic the nano-to micro-scaled fiber structure of ECM [196]. In spine engineering, electrospinning is widely used to generate ECM-like structures that replicate the AF structure. Recent advances in electrospinning for IVD engineering scaffolds focus on three key trends: the optimization of AF scaffold structures [[248], [249], [250], [251], [252], [253], [254]], the functionalization of AF scaffolds [255,256], and the application of innovative technologies [257,258].

**Fig. 9:**
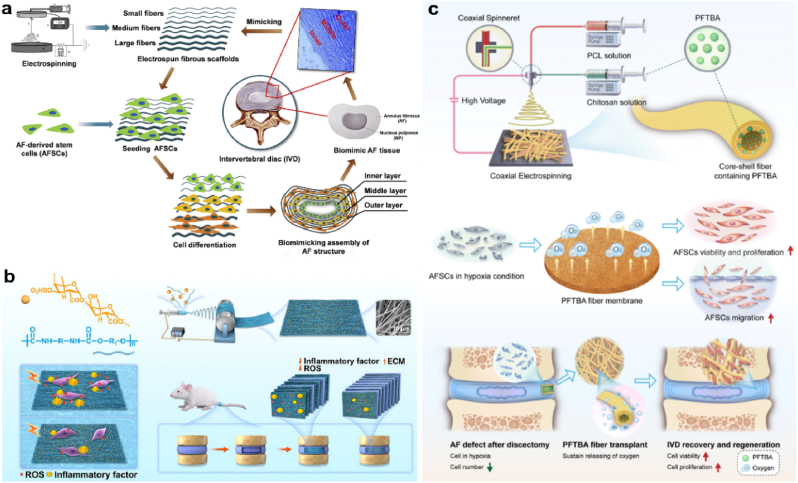
Innovations in the application of electrospinning in IVD tissue engineering. **(a)**. Schematic diagram illustrating a structurally optimized IVD tissue engineering scaffold, designed to repair AF by mimicking the hierarchical structure of native AF tissue [253]. **(b).** Schematic diagram depicting the preparation process of fucoidan-functionalized AF scaffolds, featuring anti-inflammatory and ROS-resistant nanofibers [255]. **(c).** Schematic illustration of the preparation of an AF scaffold with enhanced performance using innovative core-shell fiber electrospinning [257].

The current primary manufacturing strategy focuses on experiments to optimize the printed fiber scaffold structure with various biomaterials, aiming to replicate the structure and biomechanics of natural AF tissue, thereby aiding in the repair of damaged AF tissue. Shamsah et al. prepared electrospun filaments using different ratios of PCL/PLLA solutions and discovered that PCL/PLLA (50/50) fibers most closely resembled the structure and mechanics of AF tissue, promoting desirable cellular phenotypes [248]. After six months of *in vitro* degradation, the mechanical properties of these electrospun fiber scaffolds remained within the range reported for human AF tissue, suggesting their strength is sufficient to support AF tissue regeneration [249]. Hu et al. utilized two natural polymers, silk protein/gelatin, to prepare multilayered AF scaffolds that mimic natural AF lamellae in mechanical and morphological properties. These scaffolds induced collagen fiber tissue production within natural AF defects after 12 weeks of implantation in porcine spines, potentially enhancing AF healing [250]. Gluais et al. found that PCL scaffolds with aligned structures promoted higher levels of cell colonization, orientation, and AF-like ECM deposition both *in vitro* and *in vivo* compared to scaffolds with random fiber structures, which could serve as 3D scaffolds to induce AF-like tissue production [251]. Dewle et al. fabricated an electrospun analytically aligned PCL-supported electrically dense type I collagen patch (A-PCL-NH2+Col-I), which mimics the anisotropic, or oriented, structure of the natural mono-AF lamellae and could be used to repair and regenerate AF clefts in degenerated IVDs [252]. Another team used PLLA [253] (Fig. 9a) or poly(ester carbonate urethane)urea (PECUU) [254] to construct fiber scaffolds of different sizes and orientations, mimicking the microstructural characteristics of natural AF. These scaffolds effectively promoted the differentiation of AF stem cells (AFSCs) into specific cells similar to those found in various regions of natural AF tissue.

To improve the biological properties of AF scaffolds, another strategy is to functionalize scaffolds with bioactive factors or new biomaterials. Yu et al. developed a biocompatible PECUU nanofibrous scaffold loaded with fucoidan to endow the scaffolds with anti-inflammatory and antioxidant functions, showing good potential for AF repair in the inflammatory and oxidative microenvironment of degenerative IVDs [255] (Fig. 9b). Tu et al. prepared fiber scaffolds using basic fibroblast growth factor (bFGF), hyaluronic acid, and PLLA and incorporated type I collagen by self-assembly technique. The bFGF functionalized scaffolds enhanced the repair and regeneration of AF, and type I collagen could mimic the microenvironment of the ECM, providing structural and biochemical clues for the regeneration of AF tissue [256].

In addition, many studies focus on the innovative application of electrospinning and its combination with other biofabrication technologies in IVD tissue engineering. Yi et al. fabricated a scaffold with core-shell structured fibers by coaxial electrospinning with PCL as the shell of the fibers, chitosan and perfluorotributylamine as the core of the fibers. This design improved the oxygen release capacity of scaffolds and aided in the repair of the AF [257] (Fig. 9c). Zhu et al. combined 3D printing with electrospinning technology to construct an artificial IVD scaffold. The 3D-printed PLA skeleton offers robust mechanical support, while the electrospun PLLA fiber bundles mimic the structure of IVD tissue, and a double network hydrogel incorporated into the center of the scaffold creates a physiological environment conducive to cell loading. This integrated scaffold provides a potential candidate material for regenerating degenerated or damaged IVDs [258].

## 6 Discussion

Spinal surgery is a complex and delicate procedure, and various factors, including biologics, internal fixation systems, patient-specific factors, and surgical techniques impact its effectiveness and surgical success. Successful spinal fusion requires meticulous preparation of the decorticated vertebral endplates and/or posterolateral gutter while minimizing damage to the surrounding soft tissue. Otherwise, even using robust and expensive bone substitutes cannot compensate for a poorly prepared or devascularized fusion bed [150].

### 6.1 Autografts in spinal fusion: sustaining the gold standard amid shifting practices

Biologics play a crucial role in spinal fusion surgery by facilitating new bone formation and permanently connecting adjacent vertebrae. In clinical practice, common bone grafts include ICBGs, local autografts, allografts, DBM, and DBF. Autografts remain a favorite among surgeons due to their unique osteoconductive, osteoinductive, and osteogenic properties. ICBG is regarded as the gold standard for bone transplantation in spinal fusion surgery because of its robust osteogenic capacity and biomechanical support. However, its use has declined in recent years due to complications at the donor site [34]. Technological advancements aimed at reducing these complications may rekindle interest in ICBGs [28,31], but their limited availability continues to be a significant drawback. In contrast, local autologous transplantation is gaining increasing attention from surgeons, as it can be harvested actively or passively during decompression procedures without causing additional donor-site complications, including their effects on surgical outcomes in terms of volume [42,43] and size [45,46], along with innovative developments [38,40,[49], [50], [51], [52]]. Notably, alternative autologous graft sources such as the sternum, clavicle, and fibula are not routinely recommended due to their lack of advantages over ICBGs and donor-site complications compared to local autografts. The recently developed grafting technique of pedicled VBF [22,63], alongside the previous free VBF technique [70], can be employed to address challenging situations or cases that have failed conventional multiple bone grafting treatments. However, young surgeons often lack sufficient training in autologous transplant techniques, making transplantation of autografts more suitable for experienced surgeons. Consequently, the use of allografts and synthetic materials is gradually increasing and replacing autografts as standard practice [75].

### 6.2 Allografts and xenografts: expanding options amid standardization challenges

Allografts offer several advantages, including the absence of donor-site complications, shorter surgical time, reduced surgical blood loss, and shorter hospital stays [91,92]. They are utilized in various forms, mainly including conventional acellular mineralized allografts, cellular mineralized allografts (CBM products), DBM and DBF [81]. Innovations in these products recently arose from commercial product developments, while clinical studies primarily evaluate their effectiveness. However, a lack of standardized results exists due to inconsistencies across different manufacturers and batches due to variations in donor age, donor site, processing techniques, sterilization methods, and storage conditions [81,106]. Future research focus may be to convert the *in vitro* osteogenic potential of MSCs in CBM products into *in vivo* bone formation and to improve the delivery efficiency of DBM products by optimizing the carrier [[115], [116], [117], [118]]. Xenografts have fallen out of favor due to complications and inconsistent outcomes [120,121].

### 6.3 Bone substitutes in spinal fusion: advances in bioceramics and bioactive factors

Common bone substitutes include bioceramics like HA [124,125], BG [130,131,133], and β-TCP, as well as growth factor rhBMP-2 [150]. Recent research aims to optimize the performance of these materials by developing bioceramic variants, such as SiCaP [134,135] or bioceramic-based composites (Table 1). Polymers are often used as adhesives to provide biomechanical support [146] or as molding agents in biologics [147] to assist in spinal fusion. In the case of rhBMP-2, efforts focus on improving its delivery efficiency and reducing complications [[166], [167], [168], [169], [170]]. The labeled use of ABM/P-15 (i-Factor) in ACDF surgery [178] and its off-label use in lumbar fusion surgery [180,181] have yielded positive results. Similarly, the FDA-approved rhPDGF-BB for the fusion of the ankle and/or hindfoot has demonstrated effectiveness in preclinical studies for off-label use in lumbar fusion surgery [190,191]. However, the long-term effects and potential side effects of these treatments still require investigation through extensive studies. Given the availability and convenience of commercial products and the morbidity associated with ICBGs, it is unlikely that further randomized controlled trials will compare commercial products with ICBGs [112]. In current clinical practice, local grafts are often used and supplemented with commercial products, such as allogeneic bone or bone substitutes, when additional material is required. This approach helps avoid donor-site complications from separate incision autografts and minimizes the need for commercial products. The efficacy of many biologics is based on small sample studies conducted at single centers, which are subject to variations in surgeons' skills and assessment standards and methods. This makes it challenging to draw direct comparisons between different studies and represents a limitation in current clinical research.

### 6.4 a.m. in spinal surgery: personalized fixation and preoperative planning

New bone formation facilitated by biologics often requires time to achieve permanent osseointegration, making an internal fixation system essential for immediate stability. In this context, 3D-printed cages or AVBs can be customized to fit individual anatomical structures more precisely than traditional implants. This enhanced customization may offer a more physiologic restoration of spinal curvature and reduce complications associated with improper size. Integrating 3D-printed cage [220] or AVB [216] with fixation systems can provide significant advantages in anterior spine surgery, particularly in cervical procedures (ACDF and ACCF). Additionally, 3D printing allows for the optimization of microstructures to promote better osseointegration with the host vertebrae. 3D-printed screws also offer increased resistance to extraction, which is particularly beneficial for osteoporotic patients and potentially helps minimize screw loosening [229]. Furthermore, 3D-printed anatomical models of the spine have proven invaluable for preoperative planning, especially in complex cases involving severe deformities [206] or tumors [208]. Screw guides from 3D printing technology enable precise intraoperative screw placement in high-risk upper cervical spine surgeries [214].

### 6.5 a.m. for tissue regeneration: from bone scaffolds to disc repair

Bone regeneration scaffolds created through 3D printing support new bone formation for spinal fusion by allowing precise control over microstructures, while many applications are still in the preclinical research phase [[237], [238], [239]]. Transitioning these advantages to clinical practice requires further investigation, as the biologics used in clinical settings often differ from regular scaffolds, with putty or paste being more applicable in real-world scenarios [107]. Similarly, 3D-printed IVD scaffolds, including AF repair scaffolds [[240], [241], [242], [243]] and complete IVD replacement scaffolds [[244], [245], [246], [247]], remain under preclinical investigation. Current research focuses on regenerating AF and NP tissues *via* 3D printing or electrospinning alone or in combination. However, translating these technologies to human use presents challenges, as small animal models used [244,246] in research differ significantly from humans, who require strong mechanical support due to upright posture. Successful integration of repaired AF scaffolds with host tissue is crucial to prevent issues such as nerve compression from scaffold prolapse, and similar challenges are faced with integrating IVD scaffolds with adjacent vertebrae. Moreover, replicating IVD tissue regeneration *in vitro* or under physiological conditions with pathological variables requires further exploration. These research directions align with the growing trend towards non-fusion techniques and offer promising potential for future advancements in spinal surgery.

### 6.6 Barriers to clinical translation: challenges in 3D printing

Despite the significant promise of personalized 3D printing in reconstructing complex anatomical structures, its clinical translation and widespread adoption are still hampered by a range of challenges [259,260]. One major barrier is the time-intensive production process, which typically requires 10–12 h per model from imaging to final printing, rendering the approach impractical for emergency scenarios and high-throughput clinical environments [260]. Financial constraints also limit accessibility: the high upfront and maintenance costs of printers, software, and biomaterials, coupled with the absence of insurance reimbursement, pose substantial burdens on healthcare systems [259,260]. Additionally, implementation requires skilled personnel proficient in CAD modeling and printer operation, further raising the threshold for clinical integration. Regulatory approval remains fragmented, with most custom implants evaluated on a case-by-case basis in the absence of unified guidelines [[259], [260], [261]]. Bioprinting brings additional challenges to clinical translation. A major difficulty lies in fabricating large-scale, vascularized, and mechanically competent bone constructs within practical time and cost limits [262]. Bioprinted scaffolds often remain immature and may degrade too quickly or too slowly, either compromising structural support or triggering inflammation. While hydrogels offer excellent biocompatibility, their mechanical limitations hinder load-bearing applications. Moreover, different bioprinting modalities present technical barriers, including phototoxicity in light-based modalities, shear-induced cellular damage in extrusion-based systems, and droplet instability in inkjet-based techniques. The choice of cell sources, whether autologous, allogeneic, or stem cell-based, also affects regenerative efficacy and immunogenicity [262]. Emerging acellular strategies like exosome-loaded scaffolds show promise but remain underexplored. Additional barriers include lengthy regulatory pathways, limited clinical evidence, and modeling limitations (e.g., the STL format cannot accurately capture microstructural details) [262]. Addressing these challenges requires interdisciplinary collaboration to improve biomaterial design, streamline production workflows, and ensure their long-term safety and effectiveness through standardized testing and regulatory frameworks.

Although our review systematically summarizes the recent advancements in biologics and biomanufacturing technologies for spinal surgery, there still exist some limitations in this review. The review primarily focuses on recent developments in biologics and AM technologies within this field in the last 5 years. However, it lacks a systematic survey of each specific application from the outset, which may result in the omission of some relevant studies and detailed insights. Additionally, while the review touches on the use of stem cells in certain applications, it does not include a dedicated systematic investigation of stem cell research as other recent systematic reviews specifically address the use of stem cells in spinal fusion surgery. For more in-depth information on this topic, readers are referred to these specialized reviews [263,264].

## 7 Conclusion and future perspectives

Spinal surgery is entering a transformative era driven by innovations in biologics, biomaterials, and advanced manufacturing technologies. Autografts, particularly ICBGs and local bone grafts, remain the gold standard due to their robust osteogenic capacity, but their limitations, including limited availability and donor-site complications, have accelerated the adoption of alternatives such as allografts, DBM, synthetic bioceramics, and growth factors like rhBMP-2. Simultaneously, the application of AM technologies, especially 3D printing, is reshaping the landscape of spinal implants and intraoperative planning by enabling patient-specific cage design, anatomical modeling, and screw guide fabrication. Moreover, the integration of AM with regenerative medicine is expanding its applications to bone scaffold construction and IVD repair.

Despite recent progress, several critical and unresolved challenges remain at the forefront of biologics and AM in spinal surgery. A foremost issue lies in the standardization of biologic products, particularly CBMs and DBMs, whose bioactivity and consistency vary widely depending on donor characteristics, processing techniques, and patient-specific factors. Ensuring predictable and reproducible *in vivo* outcomes requires robust quality control frameworks and improved characterization protocols. Another major challenge involves the design of synthetic scaffolds that effectively balance osteoinductivity, mechanical strength, and controlled biodegradability, while also meeting clinical demands for scalability, safety, and cost-efficiency. The translational gap in bone and disc regeneration also persists, calling for improved *in vivo* models and biomechanical simulations that better replicate human spinal loading and pathology. On the regulatory front, existing approval pathways are poorly suited for patient-specific, AM-derived implants and bioprinted constructs, which require individualized validation processes. Finally, the integration of intraoperative AM technologies, such as real-time 3D and 4D printing, into minimally invasive spinal procedures presents both engineering and clinical challenges. These include material biocompatibility, printing resolution, surgical workflow integration, and intraoperative imaging compatibility. Addressing these barriers through interdisciplinary research will be key to translating innovation into routine practice.

Looking ahead, future research should prioritize enhancing the safety, efficacy, and clinical predictability of current biologic therapies while accelerating the development of next-generation regenerative strategies. These include stem cell-based approaches, exosome-functionalized scaffolds, and synthetic bioactive compounds with tunable release kinetics tailored to specific healing phases. Optimized delivery platforms are needed to ensure spatially and temporally precise therapeutic delivery, thereby maximizing regenerative outcomes and minimizing adverse events. In the field of spinal fixation and reconstruction, the integration of customized implants with smart materials and embedded biosensors opens up novel possibilities. These intelligent constructs can monitor local mechanical stress, implant stability, or biochemical markers in real-time, transmitting data *via* Internet-of-Things (IoT) frameworks. This enables continuous postoperative surveillance, facilitates early detection of complications, and supports dynamic treatment adjustments over the long term. The emergence of 4D printing further expands the functionality of spinal implants by introducing time-responsive behaviors. These constructs can undergo shape transformation or stiffness modulation in response to environmental cues such as temperature, pH, or inflammatory mediators. Additionally, robotic-assisted *in situ* bioprinting and photo-/acoustic-controlled ultrafine bioprinting techniques have the potential to fabricate bio-integrated structures with high precision directly at the surgical site, offering new solutions for minimally invasive and anatomically conforming tissue repair. Bridging the considerable gap between preclinical innovations and clinical routine remains a pressing challenge. This will require interdisciplinary collaboration, the implementation of multi-center longitudinal clinical trials, and the development of open-source bioengineering data ecosystems. Artificial intelligence (AI) and machine learning algorithms can further enhance this transition by supporting scaffold design optimization, outcome prediction, and real-time intraoperative decision-making.

In summary, although spinal surgery has already advanced through innovations in biologics and additive manufacturing, its next transformative leap will emerge from the convergence of medicine, biology, engineering, and data science. By fostering strategic, interdisciplinary collaboration, the field is poised to deliver increasingly personalized, intelligent, and adaptive spinal solutions, ultimately enhancing both surgical performance and long-term patient outcomes.

## CRediT authorship contribution statement

**Xinggui Tian:** Writing – review & editing, Writing – original draft, Visualization, Software, Methodology, Formal analysis, Data curation, Conceptualization. **Yakui Liu:** Writing – review & editing, Writing – original draft, Visualization, Validation, Software, Methodology, Formal analysis, Data curation, Conceptualization. **Suihong Liu:** Writing – review & editing, Visualization, Software, Conceptualization. **Qinyu Tian:** Writing – review & editing, Visualization, Software. **Deepak Bushan Raina:** Writing – review & editing, Visualization, Software, Data curation. **Michael Gelinsky:** Writing – review & editing, Supervision, Project administration, Funding acquisition. **Stefan Zwingenberger:** Writing – review & editing, Supervision, Project administration, Funding acquisition, Conceptualization.

## Informed consent statement

Not applicable.

## Institutional review board statement

Not applicable.

## Funding

This research received no external funding. Y. Liu is funded by the 10.13039/501100004543China Scholarship Council (No.202206890038), and S. Liu acknowledges support from the 10.13039/501100001809National Natural Science Foundation of China (Grant No. 32401148) and the 10.13039/501100004775Natural Science Foundation of Gansu Province, China (Grant No. 23JRRJ0004).

## Declaration of competing interest

The authors declare the following financial interests/personal relationships which may be considered as potential competing interests:D.B.R. holds stocks in Moroxite AB, Sweden. The authors declare that they have no other competing interests of this study.

## Data Availability

No data was used for the research described in the article.
